# Author Correction: General destabilizing effects of eutrophication on grassland productivity at multiple spatial scales

**DOI:** 10.1038/s41467-021-20997-9

**Published:** 2021-01-21

**Authors:** Yann Hautier, Pengfei Zhang, Michel Loreau, Kevin R. Wilcox, Eric W. Seabloom, Elizabeth T. Borer, Jarrett E. K. Byrnes, Sally E. Koerner, Kimberly J. Komatsu, Jonathan S. Lefcheck, Andy Hector, Peter B. Adler, Juan Alberti, Carlos A. Arnillas, Jonathan D. Bakker, Lars A. Brudvig, Miguel N. Bugalho, Marc Cadotte, Maria C. Caldeira, Oliver Carroll, Mick Crawley, Scott L. Collins, Pedro Daleo, Laura E. Dee, Nico Eisenhauer, Anu Eskelinen, Philip A. Fay, Benjamin Gilbert, Amandine Hansar, Forest Isbell, Johannes M. H. Knops, Andrew S. MacDougall, Rebecca L. McCulley, Joslin L. Moore, John W. Morgan, Akira S. Mori, Pablo L. Peri, Edwin T. Pos, Sally A. Power, Jodi N. Price, Peter B. Reich, Anita C. Risch, Christiane Roscher, Mahesh Sankaran, Martin Schütz, Melinda Smith, Carly Stevens, Pedro M. Tognetti, Risto Virtanen, Glenda M. Wardle, Peter A. Wilfahrt, Shaopeng Wang

**Affiliations:** 1 Ecology and Biodiversity Group, Department of Biology, Utrecht University, Padualaan 8, 3584 CH Utrecht, The Netherlands; 2 State Key Laboratory of Grassland and Agro-Ecosystems, School of Life Science, Lanzhou University, 730000 Lanzhou, Gansu Province People’s Republic of China; 3 Institute of Eco-Environmental Forensics of Shandong University, 266237 Jinan, Shandong Province People’s Republic of China; 4 Ministry of Justice Hub for Research & Practice in Eco-Environmental Forensics, 266237 Qingdao, Shandong Province People’s Republic of China; 5 Centre for Biodiversity Theory and Modelling, Theoretical and Experimental Ecology Station, CNRS, 2 route du CNRS, 09200 Moulis, France; 6 Department of Ecosystem Science and Management, University of Wyoming, Laramie, WY USA; 7 Department of Ecology, Evolution, and Behavior, University of MN, St. Paul, MN 55108 USA; 8 Department of Biology, University of Massachusetts Boston, Boston, MA 02125 USA; 9 Department of Biology, University of North Carolina Greensboro, Greensboro, NC USA; 10 Smithsonian Environmental Research Center, Edgewater, MD 21037 USA; 11 Tennenbaum Marine Observatories Network, MarineGEO, Smithsonian Environmental Research Center, Edgewater, MD 21037 USA; 12 University of Oxford Department of Plant Sciences, Oxford, OX1 3RB UK; 13 Department of Wildland Resources and the Ecology Center, Utah State University, Logan, UT 84322 USA; 14 Instituto de Investigaciones Marinas y Costeras (IIMyC), FCEyN, UNMdP-CONICET, CC 1260 Correo Central, B7600WAG Mar del Plata, Argentina; 15 Department of Physical and Environmental Sciences, University of Toronto at Scarborough, Scarborough, ON Canada; 16 School of Environmental and Forest Sciences, University of Washington, Seattle, WA 98195-4115 USA; 17 Department of Plant Biology and Program in Ecology, Evolutionary Biology, and Behavior, Michigan State University, East Lansing, MI USA; 18 Centre for Applied Ecology “Prof. Baeta Neves” (CEABN-InBIO), School of Agriculture, University of Lisbon, Lisbon, Portugal; 19 Department of Biological Sciences, University of Toronto at Scarborough, Scarborough, ON Canada; 20 Forest Research Centre, School of Agriculture, University of Lisbon, Lisbon, Portugal; 21 Department of Integrative Biology, University of Guelph, Guelph, ON N1G2W1 Canada; 22 Life Sciences, Imperial College London, Silwood Park, Ascot, SL5 7PY UK; 23 University of New Mexico, Department of Biology, Albuquerque, NM 87131 USA; 24 Department of Ecology and Evolutionary Biology, University of Colorado at Boulder, 1560 30th Street, Boulder, CO 80309-0450 USA; 25 German Centre for Integrative Biodiversity Research (iDiv) Halle-Jena-Leipzig, Deutscher Platz 5e, 04103 Leipzig, Germany; 26 Leipzig University, Institute of Biology, Deutscher Platz 5e, 04103 Leipzig, Germany; 27 Department of Physiological Diversity, Helmholtz Centre for Environmental Research - UFZ, Leipzig, Germany; 28 Department of Ecology and Genetics, University of Oulu, Oulu, Finland; 29 USDA-ARS Grassland, Soil, and Water Research Laboratory, Temple, TX 76502 USA; 30 Department of Ecology and Evolutionary Biology, University of Toronto, Toronto, ON M5S3B2 Canada; 31 Centre de recherché en écologie expérimentale et prédictive (CEREEP-Ecotron IleDeFrance), Département de biologie, Ecole normale supérieure, CNRS, PSL University, 77140 St-Pierre-les-Nemours, France; 32 Department of Heatth and Environmental Sciences, Xi’an Jiaotong liverpool University, 214123 Suzhou, Jiangsu China; 33 University of Kentucky, Plant & Soil Science, 1405 Veterans Drive, Lexington, KY 40546-0312 USA; 34 School of Biological Sciences, Monash University, Clayton Campus, Clayton, VIC 3800 Australia; 35 Department of Ecology, Environment & Evolution, La Trobe University, Bundoora, VIC 3086 Australia; 36 Graduate School of Environment and Information Sciences, Yokohama National University, 79-7 Tokiwadai, Hodogaya, Yokohama, Kanagawa 240-8501 Japan; 37 INTA (National Institute of Agricultural Research)- UNPA (Southern Patagonia National University)-CONICET, Santa Cruz, Argentina; 38 Hawkesbury Institute for the Environment, Western Sydney University, Locked Bag 1797, Penrith, NSW 2751 Australia; 39 Institute of Land, Water and Society, Charles Sturt University, Albury, NSW 2640 Australia; 40 Department of Forest Resources, University of Minnesota, Saint Paul, MN USA; 41 Swiss Federal Institute for Forest, Snow and Landscape Research WSL, Zuercherstrasse 111, 8903 Birmensdorf, Switzerland; 42 UFZ, Helmholtz Centre for Environmental Research, Physiological Diversity, Permoserstrasse 15, 04318 Leipzig, Germany; 43 Ecology & Evolution Group, National Centre for Biological Sciences, TIFR, Bangalore, Karnataka 560065 India; 44 School of Biology, University of Leeds, Leeds, LS2 9JT UK; 45 Department of Biology, Colorado State University, Fort Collins, CO 80523 USA; 46 Graduate Degree Program in Ecology, Colorado State University, Fort Collins, CO 80523 USA; 47 Lancaster Environment Centre, Lancaster University, Lancaster, LA1 4YQ UK; 48 IFEVA-Facultad de Agronomia, Universidad de Buenos Aires - CONICET, Av San Martin 4453, C1417DSE Ciudad Autonoma de Buenos Aires, Argentina; 49 School of Life and Environmental Sciences, University of Sydney, Sydney, NSW 2006 Australia; 50 Institute of Ecology, College of Urban and Environmental Science, and Key Laboratory for Earth Surface Processes of the Ministry of Education, Peking University, 100871 Beijing, China

Correction to: *Nature Communications* 10.1038/s41467-020-19252-4, published online 23 October 2020.

The original version of this Article contained an error in the author affiliations. The affiliation of Martin Schütz with Swiss Federal Institute for Forest, Snow and Landscape Research WSL, Zuercherstrasse 111, 8903 Birmensdorf, Switzerland was inadvertently omitted. Martin Schütz was incorrectly associated with Department of Forest Resources, University of Minnesota, Saint Paul, MN, US. This has now been corrected in both the PDF and HTML versions of the Article.

