# Supplementary material for: General destabilizing effects of eutrophication on grassland productivity at multiple spatial scales
Source: Nat Commun. 2020 Oct 23;11:5375. doi: 10.1038/s41467-020-19252-4 (PMC7585434; doi:10.1038/s41467-020-19252-4)
Supplement: Supplementary file 1 — Supplementary Information [file 41467_2020_19252_MOESM1_ESM.pdf]

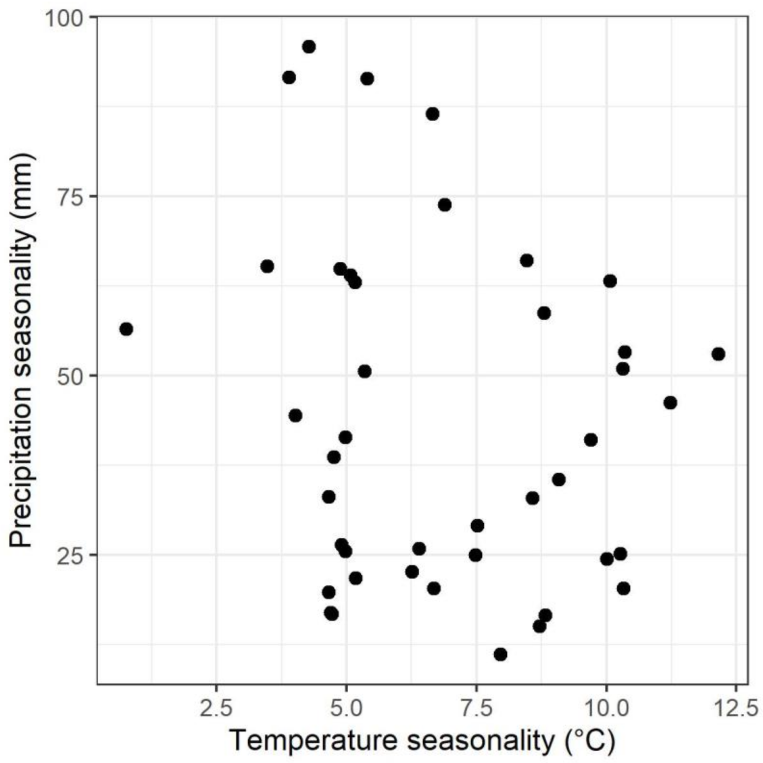

Supplementary figure 1. Study sites represent a wide range of temperature and precipitation seasonality (n=42).

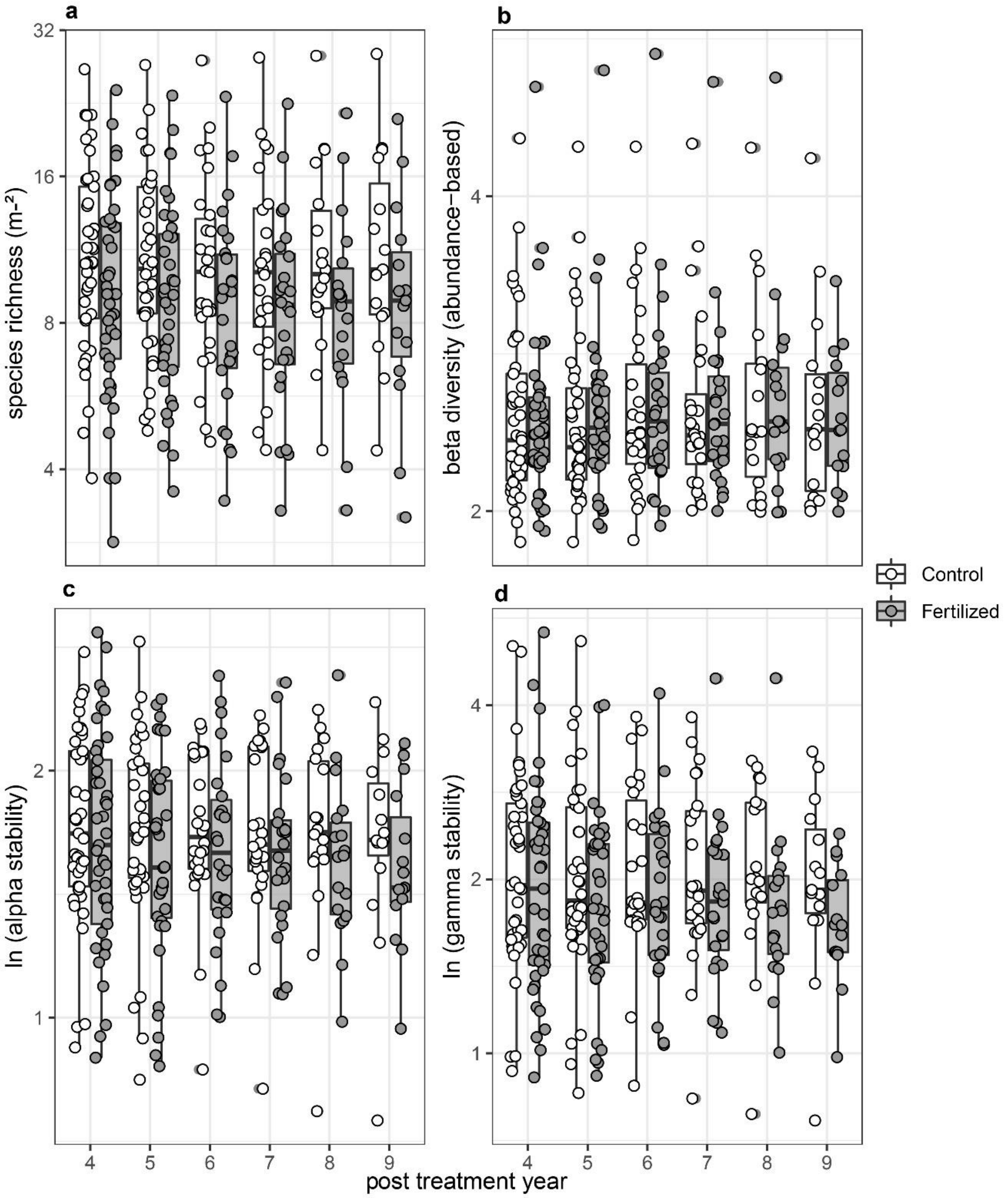

Supplementary figure 2. Impact of fertilization on diversity and stability. Analysis of variance with a first-order autoregressive model (AR(1)) determining the effect of fertilization and period of experimental duration on biodiversity and stability at the two scales investigated. a) Fertilization reduced species richness on average by -1.8 species ($F_{1, 324}$ = 82.35, P < 0.001) from 11.5 under ambient conditions to 9.7 under fertilized conditions but b) had no impact on beta diversity ($F_{1, 324}$ = 0.09, P = 0.77). c) Fertilization reduced alpha stability ($F_{1, 324}$ = 13.94, P < 0.001) from 0.75 under ambient conditions to 0.67 under fertilized conditions (log scale) and d) gamma stability ($F_{1, 324}$ = 21.81, P < 0.001) from 1.03 under ambient conditions to 0.87 under fertilized conditions (natural log transformed). Individual dots and boxes represents the median (centre line) and interquartile range (box) for the collective subplots across the three replicated 1-m^2 subplots for each site, treatment and duration period (n=160), and error bars show the 5th and 95th percentiles.

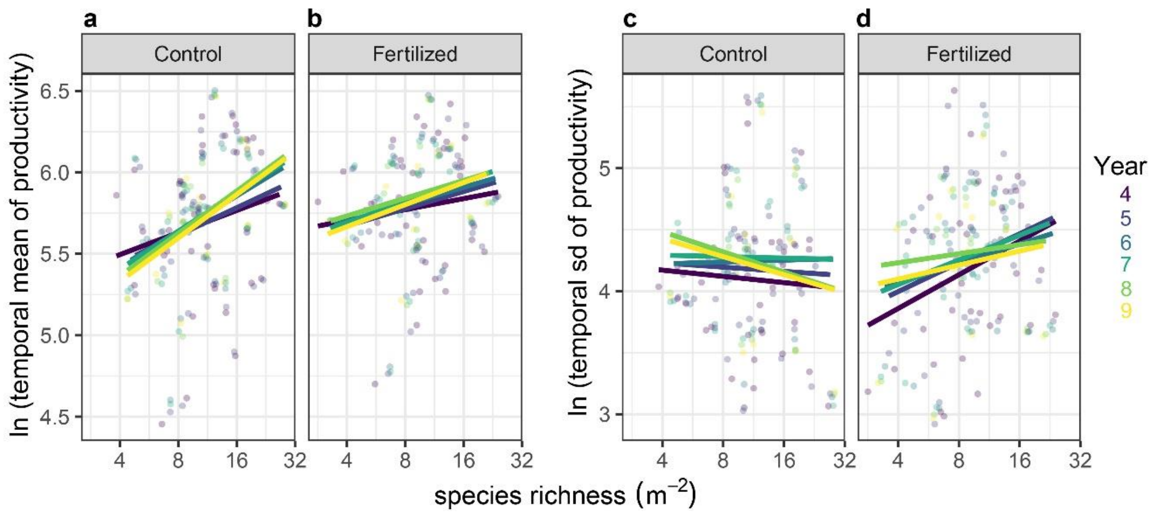

Supplementary figure 3. Impact of fertilization on the temporal mean and standard deviation of community productivity (natural log transformed). Species richness was positively associated with a) the temporal mean of community productivity in the unmanipulated communities (slope and 95% CIs across time = 0.19 (0.10 – 0.28)) and b) fertilized communities (0.10 (0.01 – 0.18)). Species richness was unrelated with c) the temporal mean of community productivity in the unmanipulated communities (-0.06 (-0.19 – 0.08)) but d) positively associated in the fertilized communities (0.20 (0.06 – 0.33)). Each dot represents the collective subplots across the three replicated 1-m^2 subplots for each site, treatment and duration period (n=160). Colours represent the periods of experimental duration.

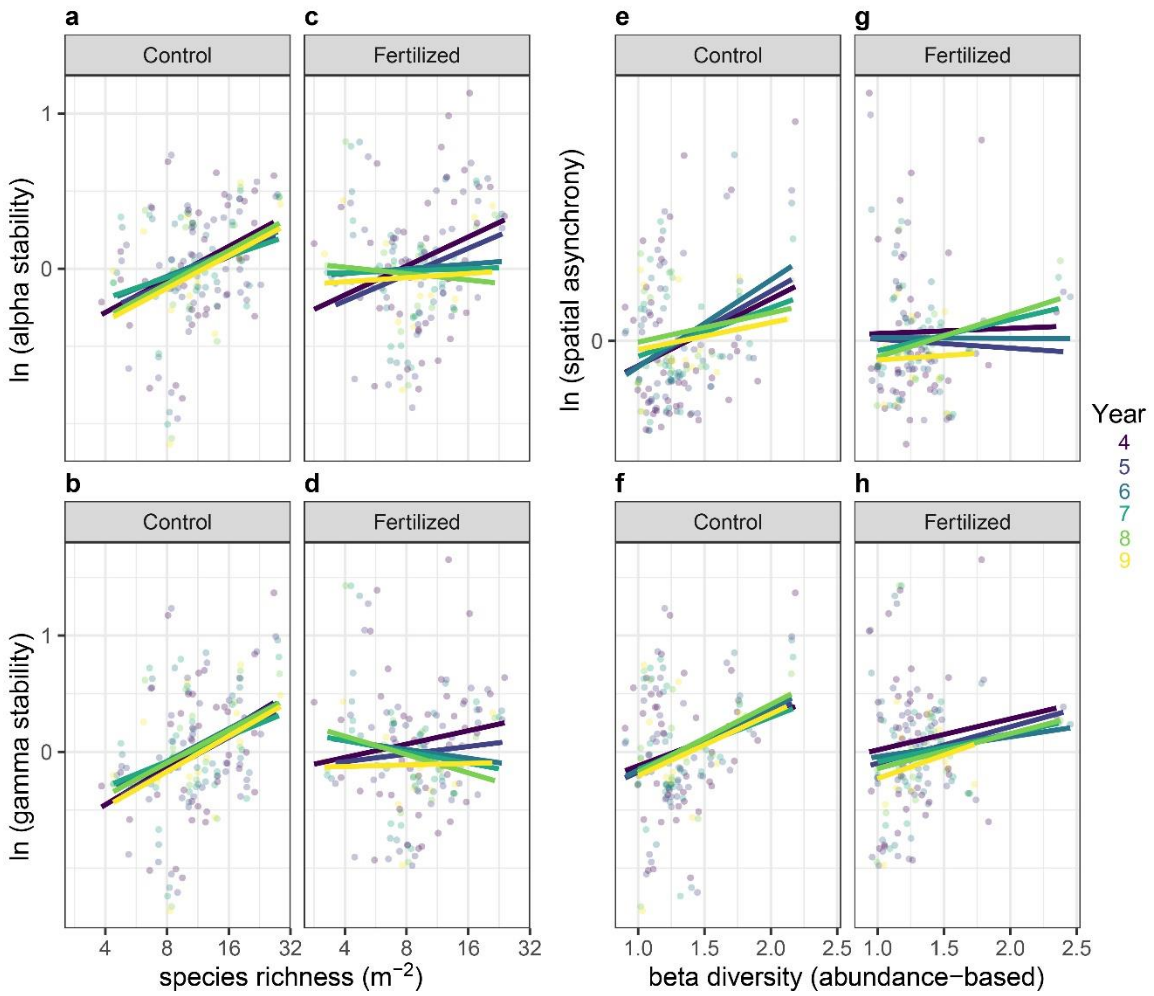

Supplementary figure 4. Impact of fertilization on biodiversity-stability relationships across spatial scales after controlling for inter-annual climate variability. The analyses was run using the residuals of models that included the coefficient of variation among years for each of temperature and precipitation. Stability was measured as the temporal mean of primary productivity divided by its temporal standard deviation. Relationships were generally consistent among the periods of experimental duration considered. Species richness was positively associated with a) alpha (slope and 95% CIs across time = 0.18 (0.10 – 0.27)) and b) gamma stability (0.28 (0.17 – 0.40)) in the unmanipulated communities, but unrelated to c) alpha (0.08 (-0.01 – 0.16)) and d) gamma stability (-0.01 (-0.10 – 0.12)) in the fertilized communities. Beta diversity was positively related to e) spatial asynchrony (0.21 (0.10 – 0.32)) and f) gamma stability (0.50 (0.23 – 0.77)) in the unmanipulated communities, but unrelated to g) spatial asynchrony (0.03 (-0.08 – 0.15)) and h) gamma stability (0.25 (-0.03 – 0.54)) in the fertilized communities. Each dot represents the collective subplots across the three replicated 1-m^2 subplots for each site, treatment and duration period (n=160). Colours represent the periods of experimental duration.

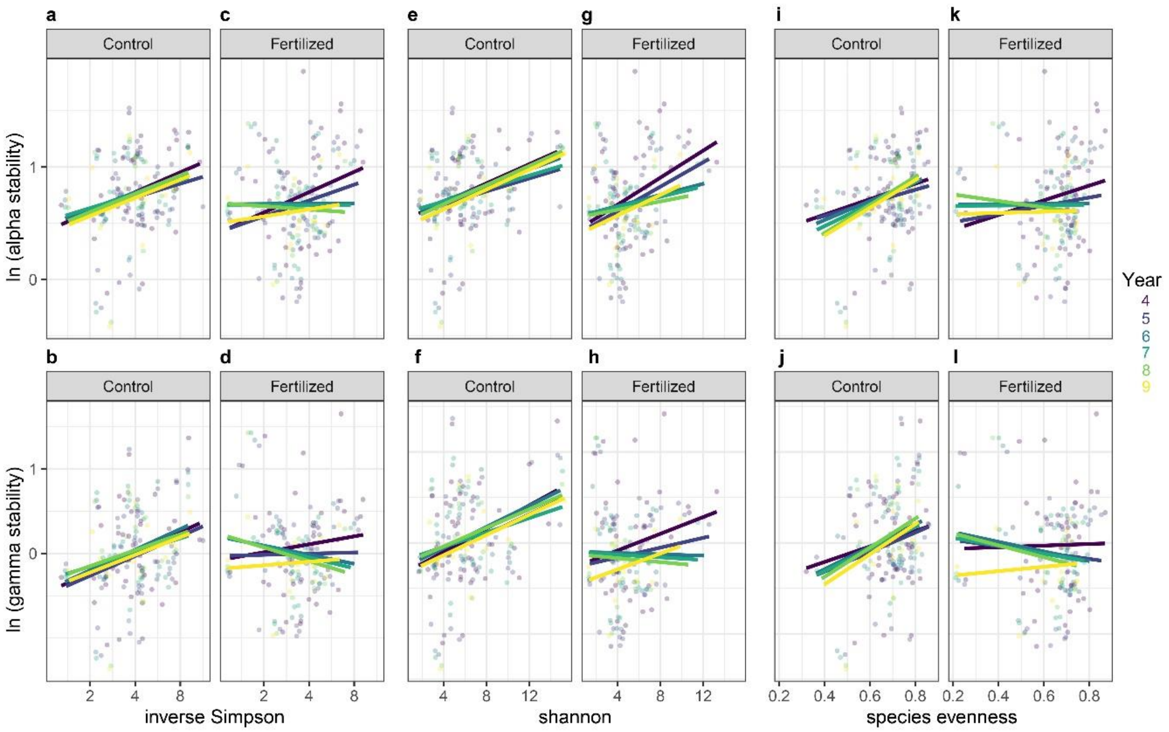

Supplementary figure 5. Impact of fertilization on biodiversity-stability relationships across spatial scales using local diversity indices accounting for species abundance. Stability was measured as the temporal mean of primary productivity divided by its temporal standard deviation. Relationships were generally consistent among the periods of experimental duration considered. Inverse Simpson was positively associated with a) alpha (slope and 95% CIs across time = 0.15 (0.06 – 0.24)) and b) gamma stability (0.22 (0.10 – 0.34)) in the unmanipulated communities, but unrelated to c) alpha (0.09 (-0.01 – 0.18)) and d) gamma stability (-0.01 (-0.13 – 0.11)) in the fertilized communities. Shannon was positively related to e) alpha (0.04 (0.02 – 0.06)) and f) gamma stability (0.06 (0.03 – 0.08)) in the unmanipulated communities, but positively related to g) alpha (0.04 (0.02 – 0.07)) and h) negatively related to gamma stability (0.02 (-0.01 – 0.05)) in the fertilized communities. Species evenness was positively related to i) alpha (0.80 (0.29 – 1.30)) and j) gamma stability (1.02 (0.32 – 1.71)) in the unmanipulated communities, but unrelated to k) alpha (0.21 (-0.21 – 0.62)) and l) gamma stability (-0.27 (-0.84 – 0.29)) in the fertilized communities. Each dot represents the collective subplots across the three replicated 1-m^2 subplots for each site, treatment and duration period (n=160). Colours represent the periods of experimental duration.

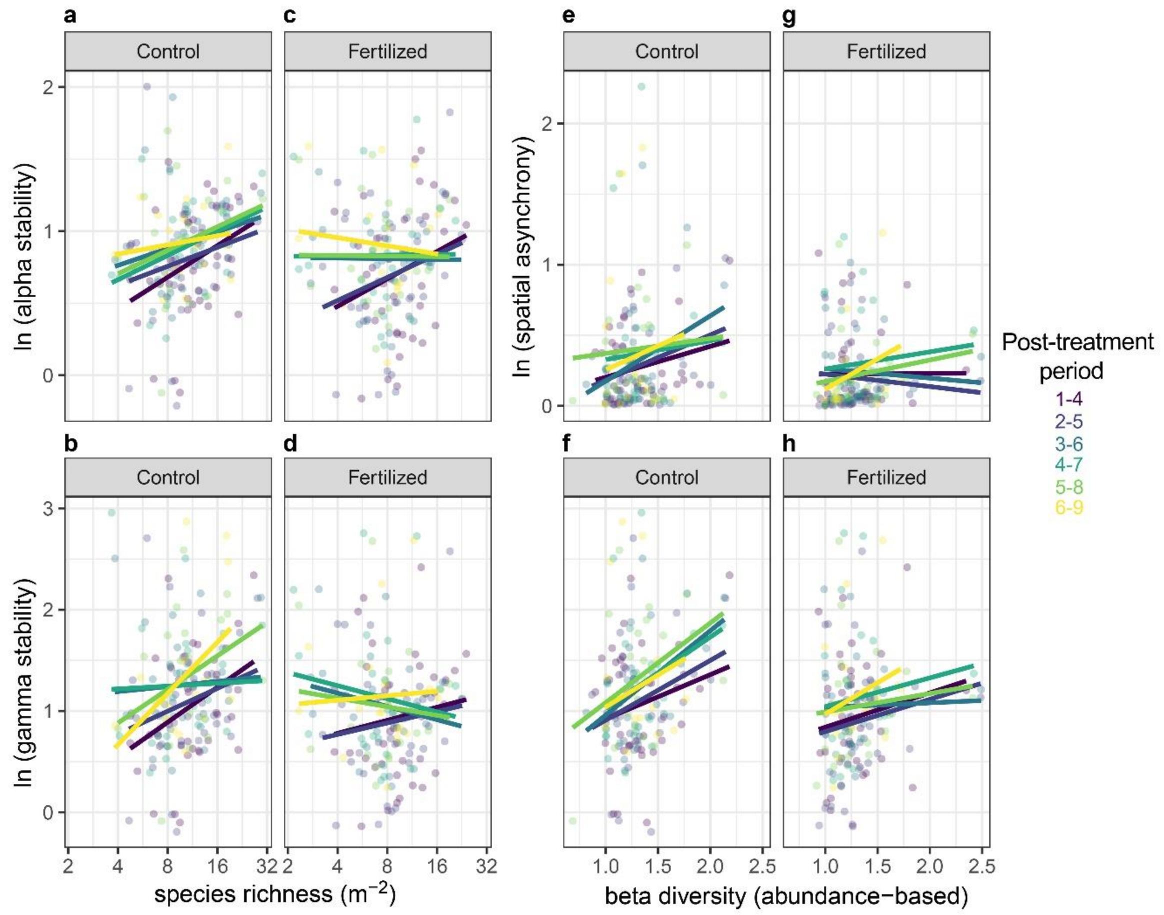

Supplementary figure 6. Impact of fertilization on biodiversity-stability relationships across spatial scales using a four years moving window. Stability was measured as the temporal mean of primary productivity divided by its temporal standard deviation. Relationships were generally consistent among the periods of experimental duration considered. Species richness was positively associated with a) alpha (slope and 95% CIs across time = 0.14 (0.05 – 0.23)) and b) gamma stability (0.21 (0.07 – 0.35)) in the unmanipulated communities, but unrelated to c) alpha (0.04 (-0.03 – 0.12)) and d) gamma stability (-0.03 (-0.15 – 0.10)) in the fertilized communities. Beta diversity was positively related to e) spatial asynchrony (0.23 (0.05 – 0.41)) and f) gamma stability (0.58 (0.27 – 0.89)) in the unmanipulated communities, but unrelated to g) spatial asynchrony (0.04 (-0.13 – 0.22)) and h) gamma stability (0.27 (-0.04 – 0.58)) in the fertilized communities. Each dot represents the collective subplots across the three replicated 1-m^2 subplots for each site, treatment and duration period (n=160). Colours represent the periods of experimental duration.

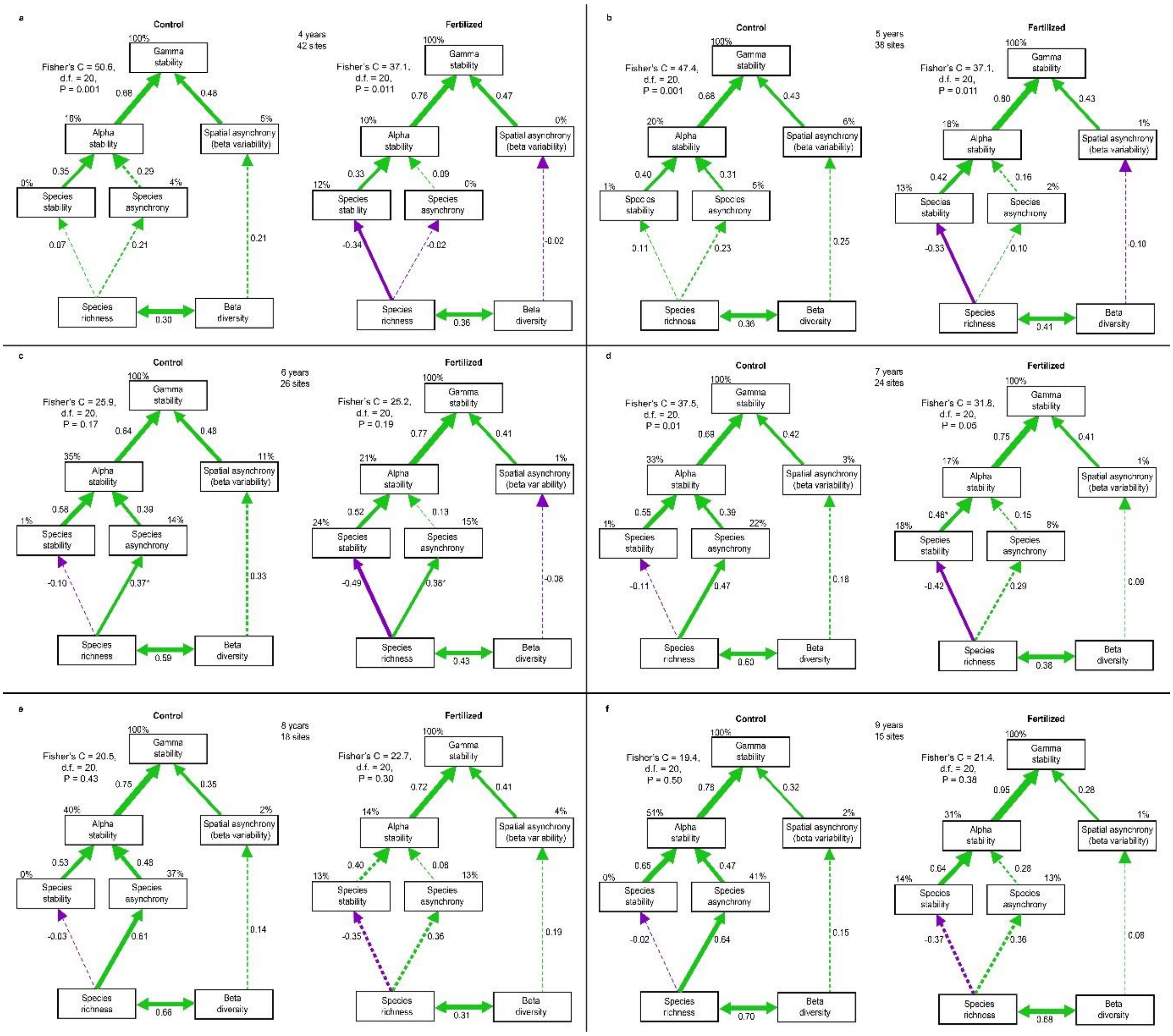

Supplementary figure 7. Structural equation modelling (SEM) showing the direct and indirect pathways through which biodiversity, asynchrony and stability at multiple spatial scales determines gamma stability. SEM model shown in Fig. 1e was evaluated separately for each period of experimental duration and treatment. Boxes represent measured variables and arrows represent relationships among variables. Numbers next to the arrows are averaged effect sizes as standardised path coefficients. Solid green and purple arrows represent significant ($P \leq 0.05$) or *marginally significant ($P \leq 0.1$) positive and negative coefficients, respectively, and dashed green and purple arrows represent non-significant coefficients. Widths of paths are scaled by standardized path coefficients. Percentages next to endogenous variables indicate the variance explained by the model (R^2). The overall model fit is given by Fisher's C statistic, which is compared to a $\chi 2$-distribution with the noted degrees of freedom. A model is considered to adequately reproduce the data if $P > 0.008$ (after Bonferroni correction for multiple testing).

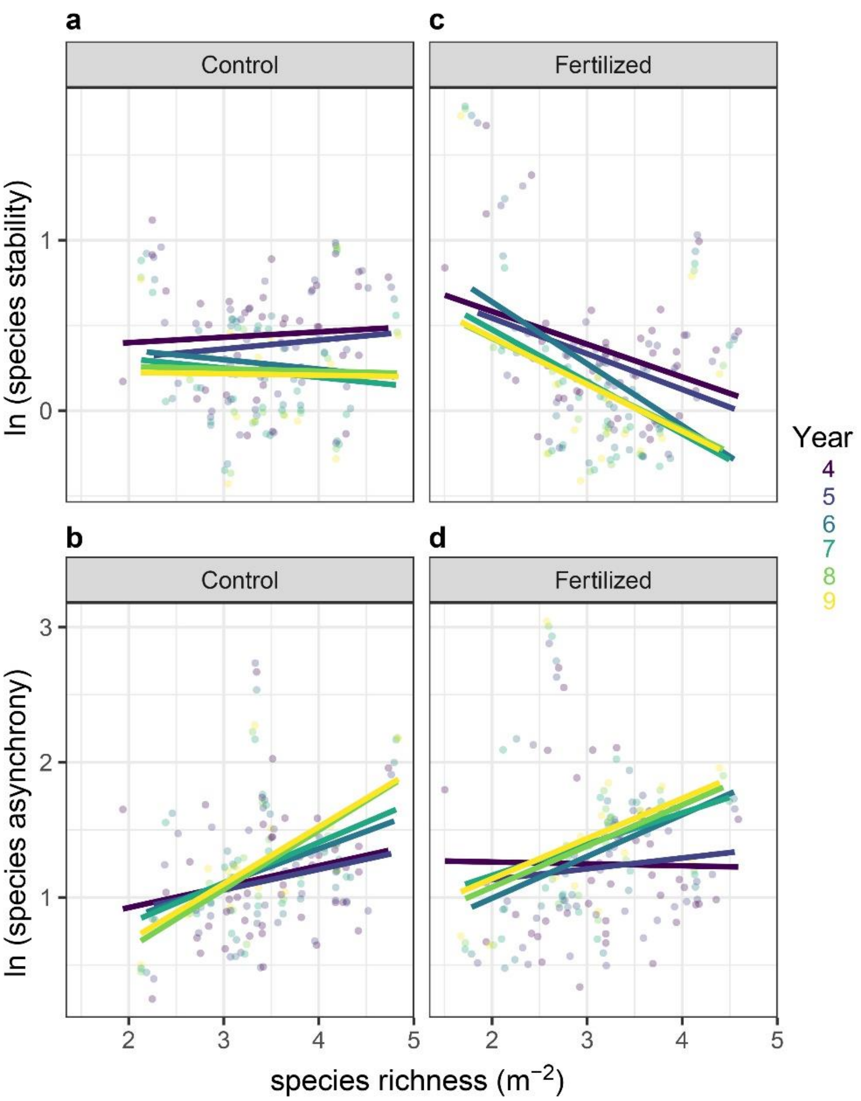

Supplementary figure 8. Impact of fertilization on the relationships between species richness and species stability (a, c) and between species richness and species asynchrony (b, d). Species stability and species asynchrony were measured using percent cover data. Relationships were generally consistent among the periods of experimental duration considered. Species richness was unrelated to a) species stability (slope and 95% CIs across time = 0.01 (-0.09 – 0.10)) and b) positively related to species asynchrony (0.24 (0.13 – 0.36)) in the unmanipulated communities, but negatively related to c) species stability (-0.24 (-0.33 – -0.15)) and d) positively related to species asynchrony (0.14 (0.03 – 0.25)) in the fertilized communities. Each dot represents the collective subplots across the three replicated 1-m^2 subplots for each site, treatment and duration period (n=160). Colours represent the periods of experimental duration.

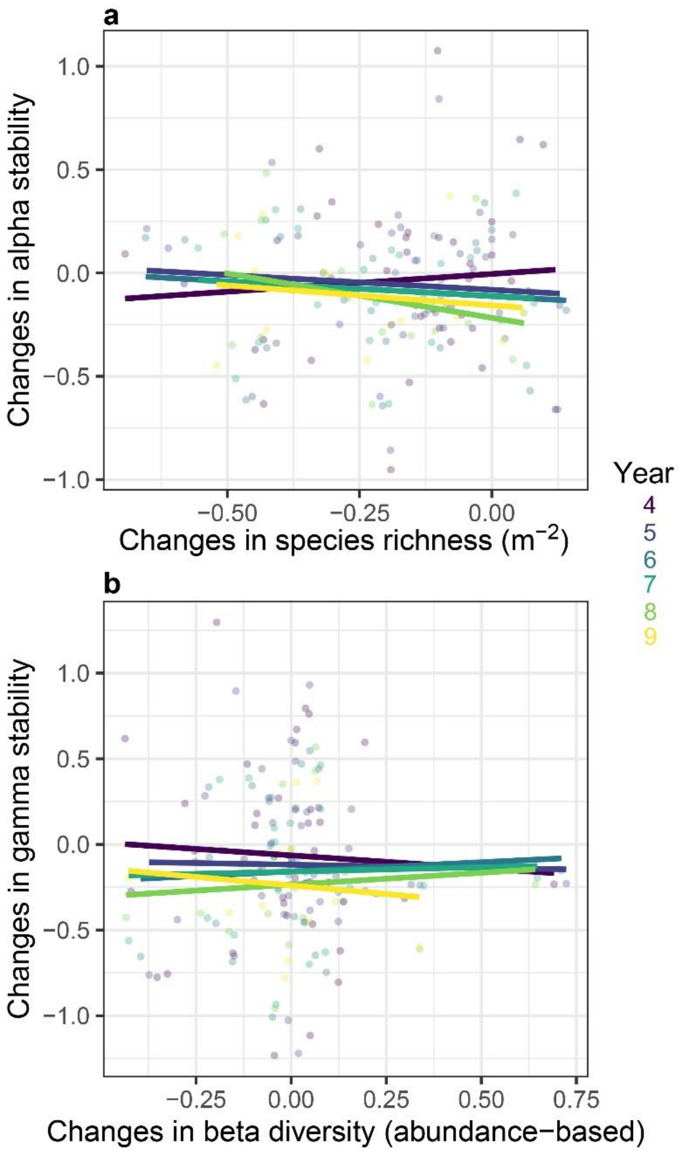

Supplementary figure 9. Effect of fertilization-induced changes in diversity on changes in stability of productivity across spatial scales. Relative changes in diversity and stability at the two scales considered were calculated as the natural logarithm of the ratio between the variable in the fertilized and unmanipulated plots. Relationships were generally consistent among the periods of experimental duration considered. a) Species richness was unrelated to alpha stability (slope and 95% CIs across time = -0.08 (-0.33 – 0.18)). b) Beta diversity was unrelated to gamma stability (-0.02 (-0.37 – 0.32)). Each dot represents the collective subplots across the three replicated 1-m^2 subplots for each site, treatment and duration period (n=160). Colours represent the periods of experimental duration.

Supplementary table 1. Anova tables for the bivariate models presented in Figure 2. ΔAIC compare AIC of models without and with a first-order autoregressive model (AR(1)). R^2 values represent the proportion of variance explained by the model. Denominator D.F. = 324 for all.

Species richness-> alpha stability<br>ΔAIC=176.7<br>R^2=0.06				Beta diversity -> spatial asynchrony<br>ΔAIC=77.1<br>R^2=0.03		
	Df	F-value	P-value	Df	F-value	P-value
(Intercept)	1	221.32	<.0001	1	119.01	<.0001
richness	1	6.93	0.001	1	0.077	0.78
time	5	0.26	0.93	5	0.264	0.93
treatment	1	8.31	0.004	1	16.52	0.0001
richness:time	5	0.57	0.73	5	0.54	0.74
richness:treatment	1	4.47	0.035	1	25.910	<.0001
time:treatment	5	1.50	0.19	5	1.16	0.32
richness:time:treatment	5	0.49	0.78	5	0.84	0.52

Species richness -> gamma stability<br>ΔAIC=166.1<br>R^2=0.07				Beta diversity -> gamma stability<br>ΔAIC=167.1<br>R^2=0.04		
	Df	F-value	P-value	Df	F-value	P-value
(Intercept)	1	221.42	<.0001	1	212.81	<.0001
richness	1	5.03	0.03	1	0.39	0.053
time	5	0.26	0.93	5	0.21	0.95
treatment	1	18.99	<.0001	1	23.85	<.0001
richness:time	5	0.38	0.86	5	0.32	0.90
richness:treatment	1	28.77	<.0001	1	23.49	<.0001
time:treatment	5	2.08	0.07	5	1.62	0.15
richness:time:treatment	5	0.18	0.97	5	0.05	0.99

Supplementary table 2. Summary of results from productivity-based meta-analysis of model paths presented in Figure 3. Show are the average effect sizes and 95% confidence intervals (CIs).

	Control		Fertilized	
Pathway	Effect size	95% CIs	Effect size	95% CIs
Richness -> Species stability	-0.02	-0.13 – 0.08	-0.40	-0.53 – -0.27
Richness -> Species asynchrony	0.42	0.29 – 0.54	0.26	0.10 – 0.41
Beta -> Spatial asynchrony	0.21	0.06 – 0.36	0.01	-0.14 – 0.16
