# Supplementary material for: General destabilizing effects of eutrophication on grassland productivity at multiple spatial scales
Source: Nat Commun. 2020 Oct 23;11:5375. doi: 10.1038/s41467-020-19252-4 (PMC7585434; doi:10.1038/s41467-020-19252-4)
Supplement: Supplementary file 3 — Description of Additional Supplementary Files [file 41467_2020_19252_MOESM3_ESM.pdf]

Description of Additonal Supplementary Files

Title: Supplementary Data 1

Description: Sites contributing experimental data. Asterisks indicate omissions based on recommendations from site leads. That is, where more than 3 blocks were established, we focused on the first three blocks unless the site lead recommended a different set of blocks.

Title: Supplementary Data 2

Description: Author contribution matrix.
