# Supplementary material for: General destabilizing effects of eutrophication on grassland productivity at multiple spatial scales
Source: Nat Commun. 2020 Oct 23;11:5375. doi: 10.1038/s41467-020-19252-4 (PMC7585434; doi:10.1038/s41467-020-19252-4)
Supplement: Supplementary file 6 — Reporting Summary [file 41467_2020_19252_MOESM6_ESM.pdf]

nature research

Corresponding author(s): Yann Hautier & Shaopeng Wang

Last updated by author(s): 16.09.2020

Reporting Summary

Nature Research wishes to improve the reproducibility of the work that we publish. This form provides structure for consistency and transparency in reporting. For further information on Nature Research policies, see our Editorial Policies and the Editorial Policy Checklist.

Statistics

For all statistical analyses, confirm that the following items are present in the figure legend, table legend, main text, or Methods section.

n/a	Confirmed	
☐	☒	The exact sample size (n) for each experimental group/condition, given as a discrete number and unit of measurement
☐	☒	A statement on whether measurements were taken from distinct samples or whether the same sample was measured repeatedly
☐	☒	The statistical test(s) used AND whether they are one- or two-sided<br>*Only common tests should be described solely by name; describe more complex techniques in the Methods section.*
☐	☒	A description of all covariates tested
☐	☒	A description of any assumptions or corrections, such as tests of normality and adjustment for multiple comparisons
☐	☒	A full description of the statistical parameters including central tendency (e.g. means) or other basic estimates (e.g. regression coefficient) AND variation (e.g. standard deviation) or associated estimates of uncertainty (e.g. confidence intervals)
☐	☒	For null hypothesis testing, the test statistic (e.g. F, t, r) with confidence intervals, effect sizes, degrees of freedom and P value noted<br>*Give P values as exact values whenever suitable.*
☒	☐	For Bayesian analysis, information on the choice of priors and Markov chain Monte Carlo settings
☐	☒	For hierarchical and complex designs, identification of the appropriate level for tests and full reporting of outcomes
☒	☐	Estimates of effect sizes (e.g. Cohen's d, Pearson's r), indicating how they were calculated

Our web collection on statistics for biologists contains articles on many of the points above.

Software and code

Policy information about availability of computer code

Data collection	Data come from the Nutrient Network, a coordinated, multi-site and multi-year nutrient enrichment experiment (+/- chronic nitrogen, phosphorus and potassium addition). Data were retrieved on 1 May 2020. The data that support the findings of this study are available via GitHub (https://github.com/YannHautier/NutNetStabilityScaleUp). No software was used for data collection
Data analysis	All analyses were conducted in R 4.0.2

For manuscripts utilizing custom algorithms or software that are central to the research but not yet described in published literature, software must be made available to editors and reviewers. We strongly encourage code deposition in a community repository (e.g. GitHub). See the Nature Research guidelines for submitting code & software for further information.

Data

Policy information about availability of data

All manuscripts must include a data availability statement. This statement should provide the following information, where applicable:

- Accession codes, unique identifiers, or web links for publicly available datasets
- A list of figures that have associated raw data
- A description of any restrictions on data availability

The data that support the findings of this study are available via GitHub (https://github.com/YannHautier/NutNetStabilityScaleUp). WorldClim global climate database is freely available through the World Data Center for Climate (WDCC; cera-www.dkrz.de), as well as through the CCAFS-Climate data portal (http://ccafs-climate.org).

April 2020

Field-specific reporting

Please select the one below that is the best fit for your research. If you are not sure, read the appropriate sections before making your selection.

☐ Life sciences ☐ Behavioural & social sciences ☒ Ecological, evolutionary & environmental sciences

For a reference copy of the document with all sections, see nature.com/documents/nr-reporting-summary-flat.pdf

Ecological, evolutionary & environmental sciences study design

All studies must disclose on these points even when the disclosure is negative.

Study description	The 42 grassland sites are part of the Nutrient Network (NutNet) Global Research Cooperative, covering a wide range of grassland habitats and relevant gradient of fine-scale and site-level variation. Treatments were randomly assigned to the plots and were replicated in three blocks at most sites (some sites had fewer/more blocks or were fully randomised).
Research sample	Plant productivity, cover, and diversity from 42 grassland sites across the globe (https://nutnet.org/).
Sampling strategy	Sampling was done in 1 m2 subplots and followed a standardized protocol at all sites. The number of samples was chosen to allow for analyses of all data, but to minimize the efforts for sampling at each site.
Data collection	Treatments and sampling followed a standardized protocol at all sites, detailed in ref. 27 (Borer, E. T. et al. Finding generality in ecology: a model for globally distributed experiments. Methods in Ecology and Evolution 5, 63-73 (2013)). Primary productivity was estimated annually by clipping at ground level using grass shears all aboveground live biomass from two 0.1 m2 (10 x 100 cm) quadrats per subplot. Areal percent cover of each species was measured concurrently with primary productivity in one 1 x 1m subplot in which no destructive sampling occurred. Cover and primary productivity were estimated twice during the year at some sites with strongly seasonal communities. We quantified local scale and larger scale diversity indices across the three replicated 1-m2 subplots for each site, treatment and duration period using cover data. Data collection was done by the principal investigator at each site.
Timing and spatial scale	We selected sites that had a minimum of four years, and up to nine years of post-treatment data. Treatment application started at most sites in 2008, but some sites started later resulting in a lower number of sites with increasing duration of the study, from 42 sites with four years of post-treatment duration to 15 sites with nine years of duration (Extended Data Table 1). Plots at all sites were 5 × 5 m (separated by at least 1 m walkways) spread over an area of at least 1000 m2. Sampling was done in 1 m2 plots grouped into spatial blocks spread over 320 m2.
Data exclusions	To keep a constant number of communities per site and treatment, we used three blocks per site, excluding additional blocks from sites that had more than three (Supplementary Table 1). We selected sites that had a minimum of four years, and up to nine years of post-treatment data. Exclusion criteria were pre-established.
Reproducibility	Data were retrieved on 1 May 2020 from the NutNet data manager. Data and codes are available via GitHub (https://github.com/YannHautier/NutNetStabilityScaleUp). Plots were replicated in three blocks at all sites used in this study.
Randomization	Treatments were randomly assigned.
Blinding	Data were analyzed using all replicates per site, but without knowing which site had which influence on the overall results.

Did the study involve field work? ☒ Yes ☐ No

Field work, collection and transport

Field conditions

Ours sites were distributed over 6 continents and 11 countries. Site name, continent, country, grassland type, latitude, longitude, period of experimental duration, average local (alpha) richness, average site (gamma) richness at each site can be found below.

Site Continent Country Type Latitude Longitude Period of experimental duration Average local (alpha) richness Average site (gamma) richness
bogong.au Australia AU alpine grassland -36.9 147.3 4,5,6,7,8,9 18.1 22.8
burrawan.au Australia AU semiarid grassland -27.7 151.1 4,5,6,7,8,9 9.3 14.8
cbgb.us America US tallgrass prairie 41.8 -93.4 4,5,6,7,8 8.4 19
cdcr.us America US tallgrass prairie 45.4 -93.2 4,5,6,7,8,9 10.6 17.8
cdpt.us America US shortgrass prairie 41.2 -101.6 4,5,6,7,8,9 11.3 20.3
chilcas.ar America AR mesic grassland -36.3 -58.3 4,5,6 10.7 23
comp.pt Europe PT annual grassland 38.8 -8.8 4,5,6,7 21.4 32.8
cowi.ca America CA old field 48.8 -123.6 4,5,6,7,8,9 4.8 7.8
elliot.us America US annual grassland 32.9 -117.1 4,5 10.7 17
frue.ch Europe CH pasture 47.1 8.5 4,5,6,7 13.9 17.8
hall.us America US tallgrass prairie 36.9 -86.7 4,5,6,7 6.3 9.8
hart.us America US shrub steppe 42.7 -119.5 4,5 8.8 14.3

hero.uk Europe UK mesic grassland 51.4 -0.6 4,5 16 21
hopl.us America US annual grassland 39 -123.1 4,5,6,7,8,9 19.4 34.8
Jena.de Europe DE grassland 50.9 11.5 4,5 18 25.5
kbs.us America US old field 42.4 -85.4 4 12.5 19.8
kilp.fi Europe FI tundra grassland 69.1 20.9 4,5 24.9 32
kiny.au Australia AU semiarid grassland -36.2 143.8 4,5,6,7,8,9 9.5 16.8
koffler.ca America CA pasture 44 -79.5 4,5,6,7,8 8.1 12.5
konz.us America US tallgrass prairie 39.1 -96.6 4,5 15.3 22.5
marc.ar America AR grassland -37.7 -57.4 4,5,6,7 11.4 20.5
mtca.au Australia AU savanna -31.8 117.6 4,5,6,7,8,9 14.1 23.8
ping.au Australia AU old field -32.5 117 4,5 7 10.5
pinj.au Australia AU pasture -27.5 152.9 4 3.8 7.3
rook.uk Europe UK mesic grassland 51.4 -0.6 4,5 9.7 12.8
saana.fi Europe FI montane grassland 69 20.8 4,5 24.7 35.3
sage.us America US montane grassland 39.4 -120.2 4,5,6 12.2 24
saline.us America US Mixed grass prairie 39.1 -99.1 4,5,6,7,8 10.6 20.3
sedg.us America US annual grassland 34.7 -120 4,5,6,7,8,9 6.8 10.8
sereng.tz Africa TZ savanna -2.3 34.5 4 12.3 20.8
sevi.us America US desert grassland 34.4 -106.7 4,5,6,7 6.3 12
sgs.us America US shortgrass prairie 40.8 -104.8 4,5,6,7,8,9 7.2 12.8
shps.us America US shrub steppe 44.2 -112.2 4,5 15.8 28
sier.us America US annual grassland 39.2 -121.3 4,5,6,7,8,9 8.1 17.5
smith.us America US mesic grassland 48.2 -122.6 4,5 19.3 26.8
spin.us America US pasture 38.1 -84.5 4,5,6,7,8,9 10.2 13.8
temple.us America US tallgrass prairie 31 -97.3 4,5,6,7,8,9 10.6 18
trel.us America US tallgrass prairie 40.1 -88.8 4,5,6,7 4.8 7
ukul.za Africa ZA mesic grassland -29.7 30.4 4,5,6,7,8,9 15.8 30.3
unc.us America US old field 36 -79 4 11.6 20
valm.ch Europe CH alpine grassland 46.6 10.4 4,5,6,7,8,9 26.6 50.8
yarra.au Australia AU mesic grassland -33.6 150.7 4,5 5.3 8.3

Location — Location (latitude and longitude) of the 42 sites is reported in Supplementary table 1 and above.

Access & import/export — Access to sites and data collection followed standard practices and complied with laws. No permits were required.

Disturbance — At each site walkways were established to minimize trampling efforts on the sampling plots

Reporting for specific materials, systems and methods

We require information from authors about some types of materials, experimental systems and methods used in many studies. Here, indicate whether each material, system or method listed is relevant to your study. If you are not sure if a list item applies to your research, read the appropriate section before selecting a response.

Materials & experimental systems

n/a	Involved in the study
☒	☐ Antibodies
☒	☐ Eukaryotic cell lines
☒	☐ Palaeontology and archaeology
☒	☐ Animals and other organisms
☒	☐ Human research participants
☒	☐ Clinical data
☒	☐ Dual use research of concern

Methods

n/a	Involved in the study
☒	☐ ChIP-seq
☒	☐ Flow cytometry
☒	☐ MRI-based neuroimaging
